# 

**Published:** 1871-07

**Authors:** 


Vol. I. July, 1871. No. 7.

### THE GEORGIA MEDICAL COMPANION,

DEVOTED TO THE

### SCIENCE OF MEDICINE AND SURGERY.

EDITORS:

T. S. POWELL, M. D., and W. T. GOLDSMITH, M. D.

ASSOCIATE EDITORS:

ROBERT BATTEY, M. D., Rome, Ga.; R. C. WORD, M. D., Tunnel Hill, Ga.; JAMES A. LONG, M. D, LaGrange, Ga.; E. F. KNOTT, M. D., Griffin, Ga.; W. L. M. HARRIS, M. D., Greensboro, Ga.; W. C. MUSGROVE, M. D., Midville, Ga.; C. H. BASS, M. D., Lunatic Asylum, Ga.; JOHN C. DRAKE, M. D., Thomaston, Ga.; A. H. BRANTLY, M. D., Decatur, Ga.; J. J. KNOTT, M. D., Atlanta, Ga.

*"QUICQUID PRÆCIPIES ESTO BREVIS."*

ATLANTA, GA.

Franklin Steam Printing House.

J. J. TOON, Proprietor.

Terms, - - - - $2 a Year, in Advance.

